# Supplementary figures and images for: Assessing fetal growth in Africa: Application of the international WHO and INTERGROWTH-21st standards in a Beninese pregnancy cohort
Source: PLoS One. 2022 Jan 21;17(1):e0262760. doi: 10.1371/journal.pone.0262760 (PMC8782373; doi:10.1371/journal.pone.0262760)

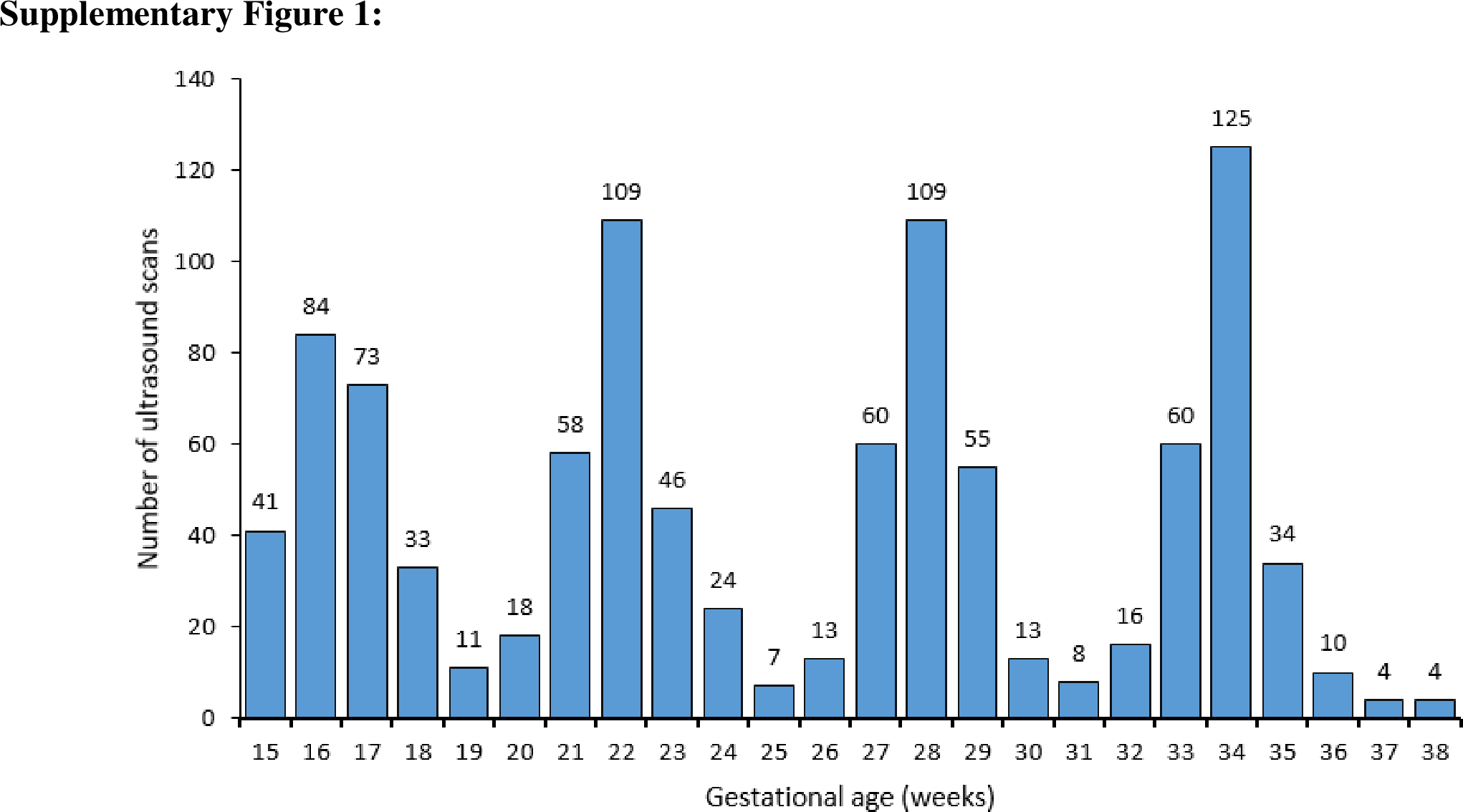

Supplement: S1 Fig — RECIPAL cohort, Southern Benin, 2014–2017. (TIF) [file pone.0262760.s001.tif]

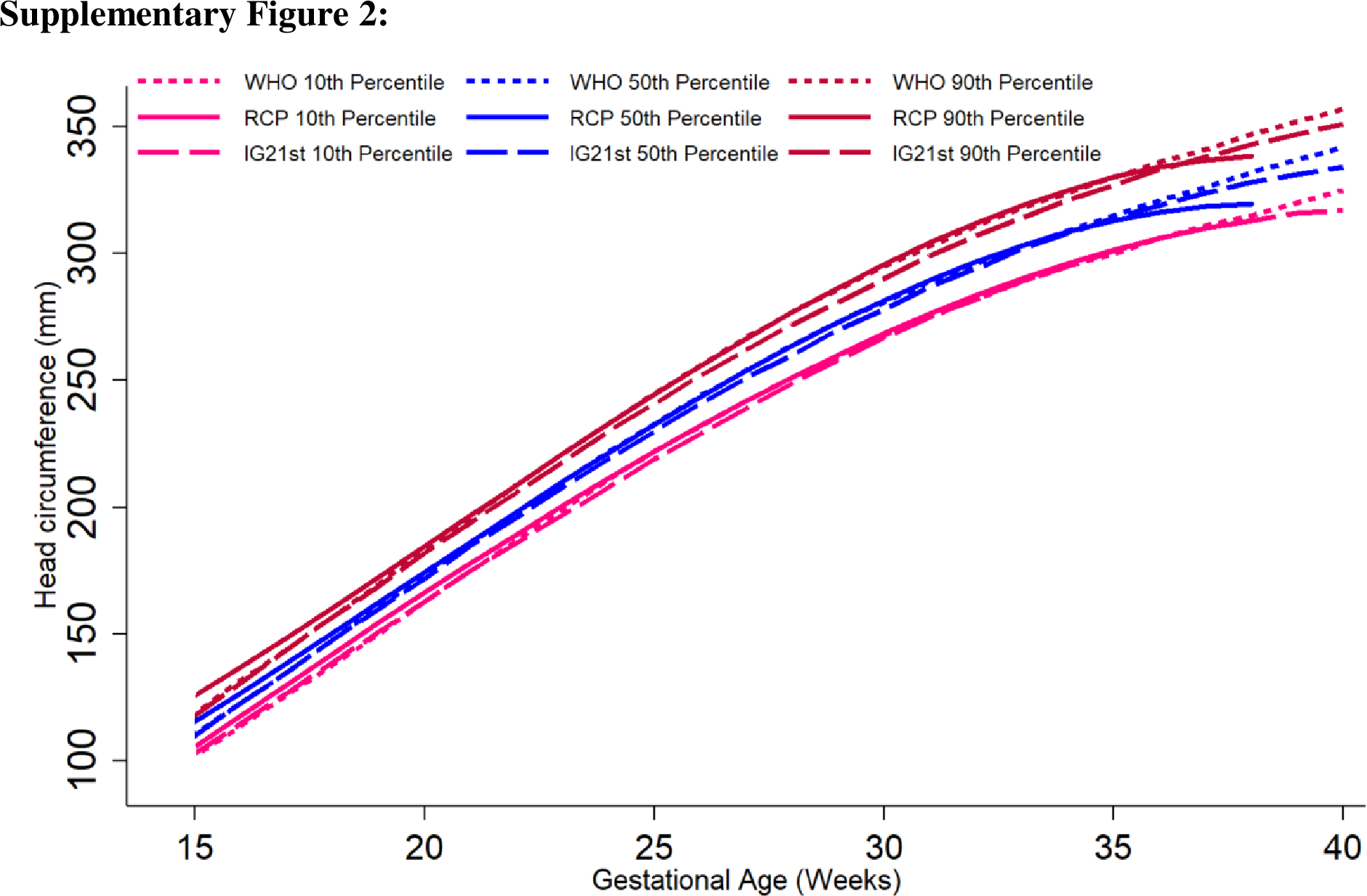

Supplement: S2 Fig — RECIPAL cohort, Southern Benin, 2014–2017. (TIF) [file pone.0262760.s002.tif]

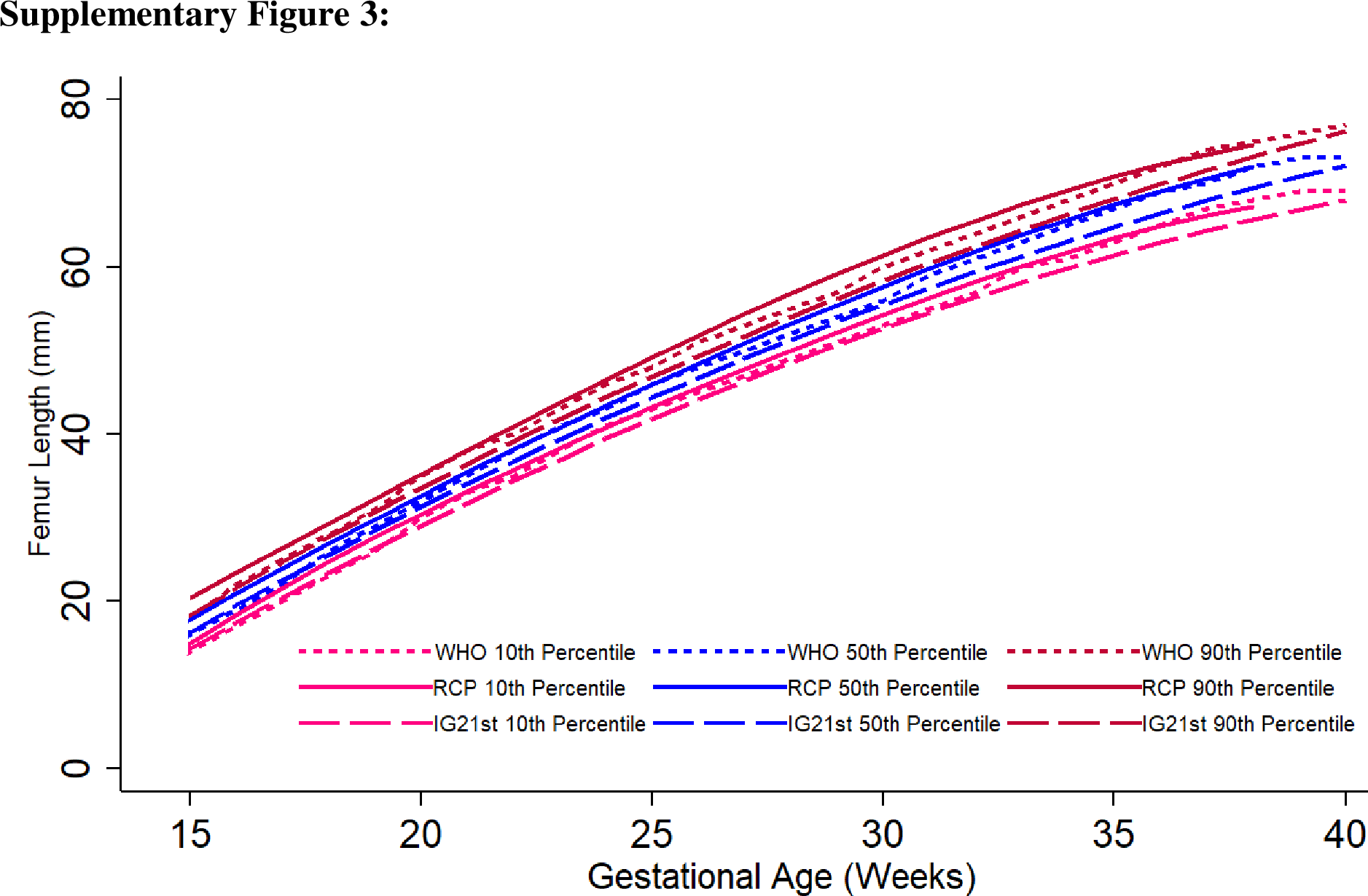

Supplement: S3 Fig — RECIPAL cohort, Southern Benin, 2014–2017. (TIF) [file pone.0262760.s003.tif]

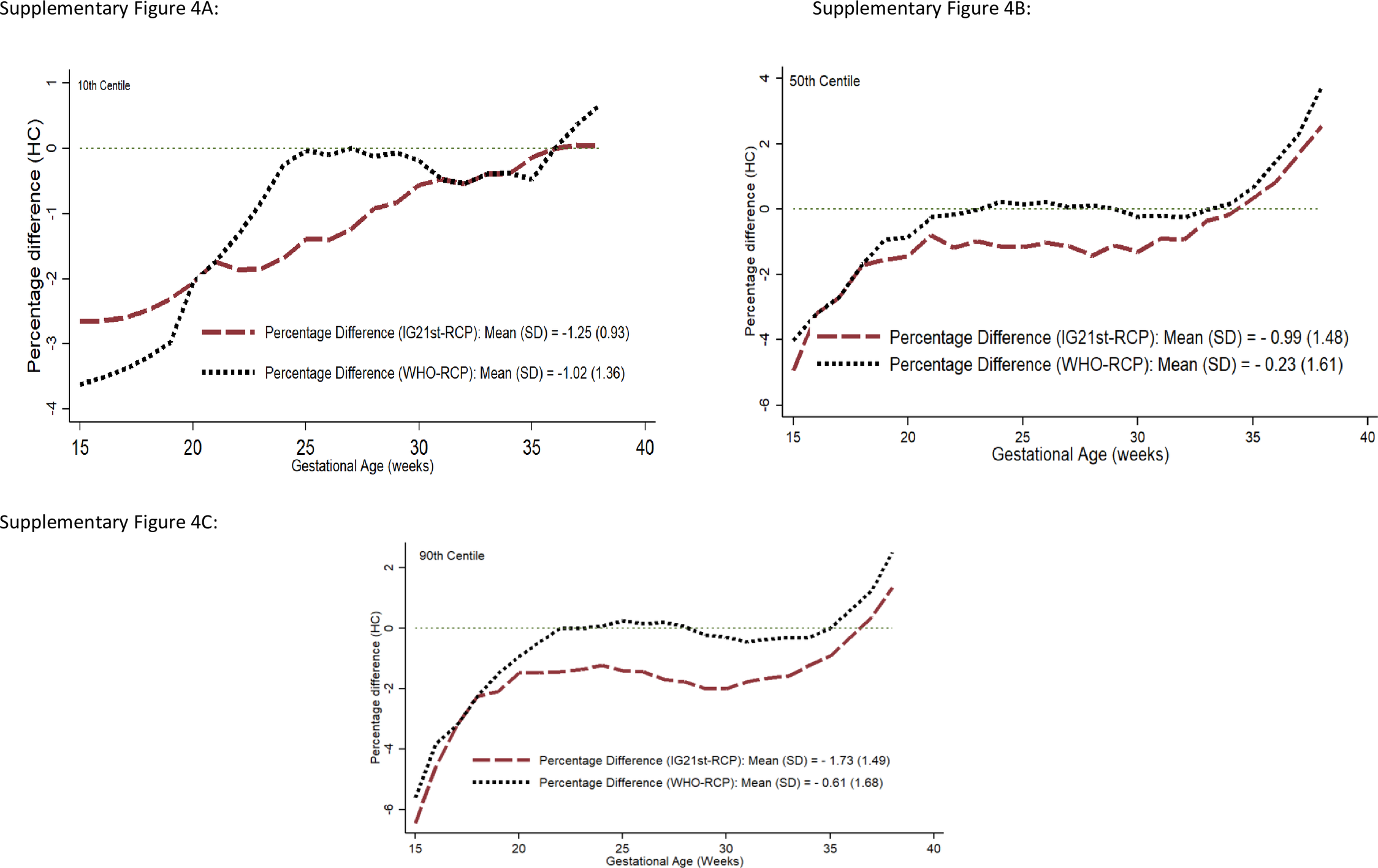

Supplement: S4 Fig — Percentage difference in 10th (A), 50th (B), and 90th (C) centiles of head circumference (HC) between RECIPAL centiles and those of WHO and INTERGROWTH-21st. RECIPAL cohort, Southern Benin, 2014–2017. (TIF) [file pone.0262760.s004.tif]

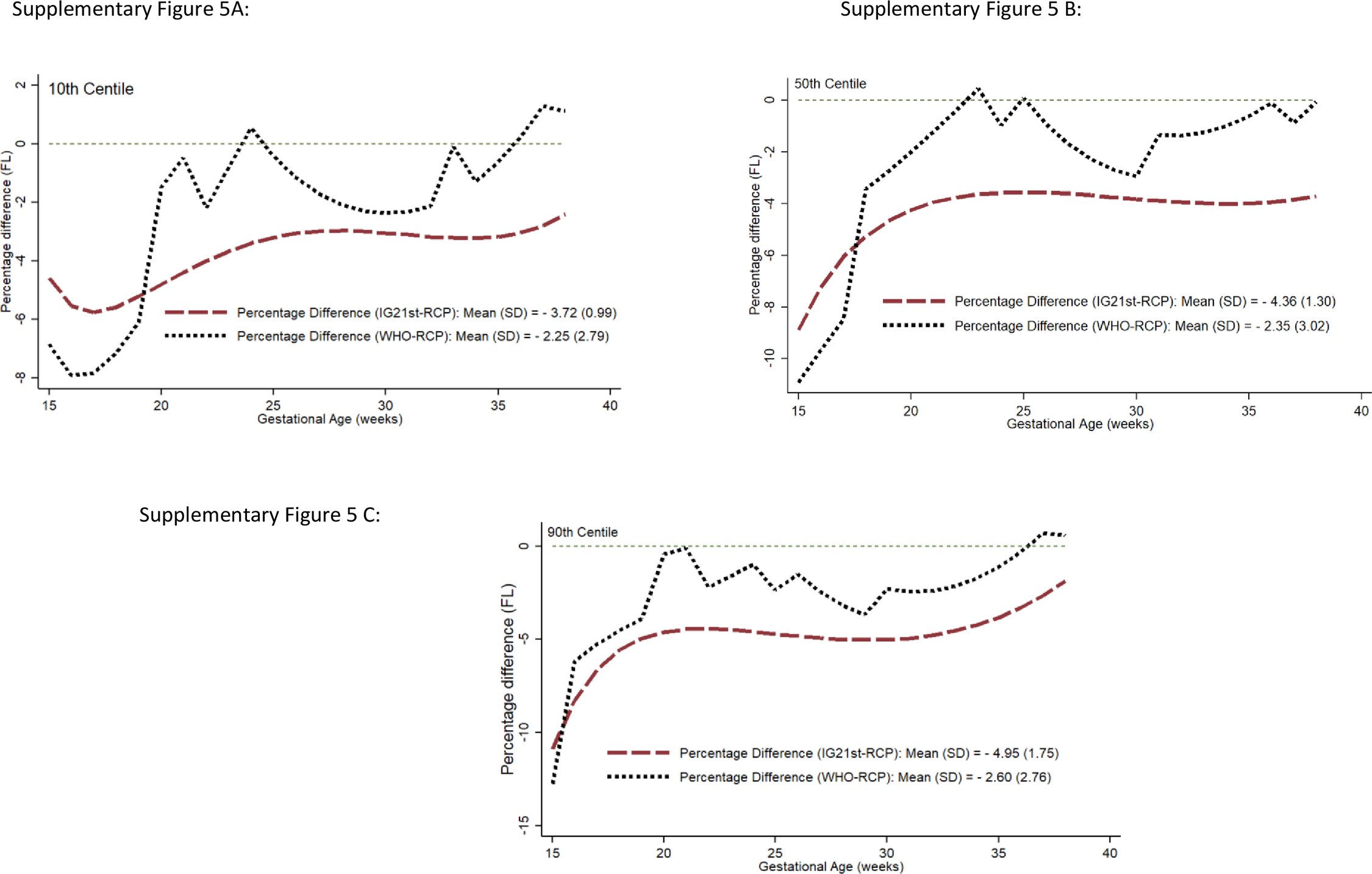

Supplement: S5 Fig — Percentage difference in 10th (A), 50th (B), and 90th (C) centiles of femur length (FL) between RECIPAL centiles and those of WHO and INTERGROWTH-21st. RECIPAL cohort, Southern Benin, 2014–2017. (TIF) [file pone.0262760.s005.tif]
