# Supplementary material for: Assessing fetal growth in Africa: Application of the international WHO and INTERGROWTH-21st standards in a Beninese pregnancy cohort
Source: PLoS One. 2022 Jan 21;17(1):e0262760. doi: 10.1371/journal.pone.0262760 (PMC8782373; doi:10.1371/journal.pone.0262760)
Supplement: S1 File — Supplementary Table S1: Equations for the estimation of each percentile using quantile regression of each fetal measurement (in mm) according to gestational age (in weeks). Supplementary Table S2: Centiles of abdominal circumference (N = 241). RECIPAL cohort, Southern Benin, 2014–2017. Supplementary Table S3: Centiles of head circumference (N = 241). RECIPAL cohort, Southern Benin, 2014–2017. Supplementary Table S4: Centiles of estimated fetal weight using the Hadlock formula (N = 241). RECIPAL cohort, Southern Benin, 2014–2017. Supplementary Table S5: Centiles of estimated fetal weight using the INTERGROWTH-21st formula starting from 22 weeks of gestation (N = 241). RECIPAL cohort, Southern Benin, 2014–2017. Supplementary Table S6: Centiles of femur length (N = 241). RECIPAL cohort, Southern Benin, 2014–2017. Supplementary Table S7: The proportion of fetuses with observed values below the threshold of each percentile (Q) using quantile regression to model RECIPAL data, RECIPAL cohort, Southern Benin, 2014–2017. (DOCX) [file pone.0262760.s006.docx]

**Supplementary Table S1:** Equations for the estimation of each percentile using quantile regression of each fetal measurement (in mm) according to gestational age (in weeks)

| **Abdominal Circumference** | | | |
| --- | --- | --- | --- |
| Q3: -130.0273 | +16.99991×GA | -0.2338235× GA^2^ | + 0.0023619× GA^3^ |
| Q5: -36.54372 | +5.592837×GA | +0.2204179× GA^2^ | -0.0034103× GA^3^ |
| Q10: -59.94001 | +8.283316×GA | +0.1348214× GA^2^ | -0.0024969× GA^3^ |
| Q25: -17.75032 | +2.979228×GA | +0.3639512× GA^2^ | -0.0054895× GA^3^ |
| Q50: -2.90625 | +1.568119×GA | +0.423085× GA^2^ | -0.0061658× GA^3^ |
| Q75: 25.15594 | -1.483168×GA | +0.5496941× GA^2^ | -0.0077263× GA^3^ |
| Q90: -33.94444 | +6.5×GA | +0.2291667× GA^2^ | -0.0034722× GA^3^ |
| Q95: -41.14286 | +7.846429×GA | +0.1741071× GA^2^ | -0.0026786× GA^3^ |
| Q97: 58.48393 | -5.467065×GA | +0.7480563× GA^2^ | -0.0104279× GA^3^ |
| *GA = Gestational Age (ranging 15-38 weeks)* | | | |

| **Head Circumference** |
| --- |
| \| Q3: -46.94524 \| 6.035504×GA \| 0.3702381× GA^2^ \| -0.007563× GA^3^ \| \| --- \| --- \| --- \| --- \| \| Q5: -43.52392 \| 6.242575×GA \| 0.3481929× GA^2^ \| -0.0070788× GA^3^ \| \| Q10: -63.10714 \| 8.895833×GA \| 0.2430556× GA^2^ \| -0.0057044× GA^3^ \| \| Q25: -54.06122 \| 7.62568×GA \| 0.317432× GA^2^ \| -0.0068878× GA^3^ \| \| Q50: 18.76313 \| -0.9326313×GA \| 0.6615537× GA^2^ \| -0.011279× GA^3^ \| \| Q75: 33.71894 \| -1.953511×GA \| 0.6935342× GA^2^ \| -0.011488× GA^3^ \| \| Q90: 45.09109 \| -3.032148×GA \| 0.7426258× GA^2^ \| -0.0120976× GA^3^ \| \| Q95: 83.86471 \| -7.83493×GA \| 0.9538655× GA^2^ \| -0.01507× GA^3^ \| \| Q97: 46.16029 \| -2.548374×GA \| 0.7267601× GA^2^ \| -0.0119475× GA^3^ \| \| *GA = Gestational Age (ranging 15-38 weeks)* \| \| \| \| |

| **Femur Length** |
| --- |
| \| Q3: 16.874 \| -5.786183×GA \| 0.5933715× GA^2^ \| -0.0169784× GA3 \| 0.0001655× GA^4^ \| \| --- \| --- \| --- \| --- \| --- \| \| Q5: -58.6668 \| 7.109419×GA \| -0.2027803× GA^2^ \| 0.0042276× GA3 \| -0.0000403× GA^4^ ² \| \| Q10: -79.7551 \| 10.7237×GA \| -0.4215092× GA^2^ \| 0.0099265× GA^3^ \| -0.0000943× GA^4^ \| \| Q25: -87.68345 \| 12.23233×GA \| -0.5170813× GA^2^ \| 0.0126402× GA^3^ \| -0.0001225× GA^4^ \| \| Q50: -38.28646 \| 4.57785×GA \| -0.0734461× GA^2^ \| 0.001451× GA^3^ \| -0.0000178× GA^4^ \| \| Q75: -20.29187 \| 1.504705×GA \| 0.1205009× GA^2^ \| -0.0035702× GA^3^ \| 0.0000281× GA^4^ \| \| Q90: -18.41142 \| 1.983229×GA \| 0.056486× GA^2^ \| -0.0009997× GA^3^ \| -4.36E-06× GA^4^ \| \| Q95: -42.83034 \| 6.369389×GA \| -0.2182179× GA^2^ \| 0.0063363× GA^3^ \| -0.0000751× GA^4^ \| \| Q97: -72.75679 \| 11.08503×GA \| -0.4829888× GA^2^ \| 0.0128139× GA^3^ \| -0.0001335× GA^4^ \| \| *GA = Gestational Age (ranging 15-38 weeks)* \| \| \| \| \| |

| **Estimated fetal weight (using Hadlock formula)** |
| --- |
| Q3: 343.2216 -41.07431×GA + 0.9823108 × GA^2^ + 0.0447881× GA^3^ |
| Q5: 462.1366 -56.74229×GA + 1.608397× GA^2^ + 0.0384806× GA^3^ |
| Q10: 597.5086 -77.62477×GA + 2.621996× GA^2^ + 0.0249399× GA^3^ |
| Q25: 1408.786 -189.0114×GA + 7.470553× GA^2^ - 0.0389549× GA^3^ |
| Q50: 1124.862 -145.4908×GA + 5.292908× GA^2^ - 0.0002946× GA^3^ |
| Q75: 1420.291 -185.0366×GA + 6.930129× GA^2^ - 0.0170559× GA^3^ |
| Q90: 1510.977 -193.1168×GA + 7.040168× GA^2^ - 0.0105062× GA^3^ |
| Q95: 1415.892 -182.4964×GA + 6.650224× GA^2^ - 0.0028826× GA^3^ |
| Q97: 1376.208 -183.1296×GA + 6.963783× GA^2^ - 0.0090821× GA^3^ |
| *GA = Gestational Age (ranging 15-38 weeks)* |

| **Estimated fetal weight (using INTERGROWTH-21st formula)** |
| --- |
| \| \| Q3: 24.69139 + 34.45719 × AG -2.636394 × GA^2^ + 0.0899354 × GA^3^ \| \| --- \| \| Q5: -42.78551 + 45.39588 × AG -3.254431 × GA^2^ + 0.1027094 × GA^3^ \| \| Q10: 293.0006 - 2.706951 × AG -1.072274 × GA^2^ + 0.0727025 × GA^3^ \| \| Q25: 666.7384 - 52.4621 × AG + 0.9787968 × GA^2^ + 0.0496481 × GA^3^ \| \| Q50: 726.3279 - 54.89478 × AG + 0.7287928 × GA^2^ + 0.0625468 × GA^3^ \| \| Q75: 655.6087 - 43.04047 × AG + 0.0279552 × GA^2^ + 0.0797539 × GA^3^ \| \| Q90: 916.2337 -77.11522 × AG + 1.36487 × GA^2^ + 0.0685149 × GA^3^ \| \| Q95: 1345.061 -132.6934 × AG + 3.61296 × GA^2^ + 0.0426334 × GA^3^ \| \| Q97: 809.9163 -55.78485 × AG + -0.017256 × GA^2^ + 0.1004389 × GA^3^ \| \| \| --- \| --- \| --- \| --- \| --- \| --- \| --- \| --- \| --- \| --- \| |
| *GA = Gestational Age (ranging 15-38 weeks)* |

**Supplementary Table S2:** Centiles of abdominal circumference (N=241). RECIPAL cohort, Southern Benin, 2014-2017.

| Week of gestation |  | 3th | 5th | 10th | 50th | 90th | 95th | 97th |
| --- | --- | --- | --- | --- | --- | --- | --- | --- |
| 15 |  | 80 | 85 | 86 | 95 | 103 | 107 | 110 |
| 16 |  | 92 | 95 | 97 | 105 | 115 | 118 | 120 |
| 17 |  | 103 | 105 | 108 | 116 | 126 | 129 | 131 |
| 18 |  | 114 | 116 | 118 | 126 | 137 | 141 | 142 |
| 19 |  | 125 | 126 | 129 | 137 | 148 | 152 | 153 |
| 20 |  | 135 | 136 | 140 | 148 | 160 | 164 | 165 |
| 21 |  | 146 | 147 | 150 | 160 | 171 | 176 | 177 |
| 22 |  | 156 | 157 | 161 | 171 | 183 | 187 | 189 |
| 23 |  | 166 | 167 | 172 | 182 | 195 | 199 | 202 |
| 24 |  | 176 | 178 | 182 | 193 | 206 | 210 | 214 |
| 25 |  | 186 | 188 | 192 | 204 | 218 | 222 | 226 |
| 26 |  | 195 | 198 | 203 | 215 | 229 | 233 | 239 |
| 27 |  | 205 | 208 | 213 | 227 | 240 | 245 | 251 |
| 28 |  | 215 | 218 | 223 | 237 | 252 | 256 | 263 |
| 29 |  | 224 | 228 | 233 | 248 | 263 | 268 | 275 |
| 30 |  | 233 | 238 | 242 | 258 | 274 | 279 | 286 |
| 31 |  | 243 | 247 | 252 | 269 | 284 | 290 | 297 |
| 32 |  | 252 | 256 | 261 | 278 | 295 | 300 | 308 |
| 33 |  | 261 | 265 | 271 | 288 | 305 | 311 | 318 |
| 34 |  | 271 | 274 | 279 | 297 | 316 | 322 | 328 |
| 35 |  | 280 | 283 | 288 | 306 | 325 | 332 | 336 |
| 36 |  | 289 | 291 | 296 | 314 | 335 | 342 | 345 |
| 37 |  | 299 | 299 | 305 | 322 | 344 | 352 | 352 |
| 38 |  | 308 | 307 | 313 | 329 | 353 | 361 | 359 |

**Supplementary Table S3:** Centiles of head circumference (N=241). RECIPAL cohort, Southern Benin, 2014-2017.

| Week of gestation | 3th | 5th | 10th | 50th | 90th | 95th | 97th |
| --- | --- | --- | --- | --- | --- | --- | --- |
| 15 | 101 | 105 | 106 | 116 | 126 | 130 | 131 |
| 16 | 113 | 117 | 118 | 127 | 137 | 141 | 143 |
| 17 | 126 | 128 | 130 | 139 | 149 | 152 | 154 |
| 18 | 138 | 140 | 143 | 151 | 161 | 164 | 166 |
| 19 | 150 | 152 | 155 | 163 | 173 | 176 | 178 |
| 20 | 161 | 164 | 166 | 175 | 185 | 188 | 190 |
| 21 | 173 | 176 | 178 | 186 | 197 | 200 | 203 |
| 22 | 185 | 187 | 190 | 198 | 209 | 213 | 215 |
| 23 | 196 | 198 | 201 | 210 | 221 | 225 | 227 |
| 24 | 207 | 209 | 212 | 222 | 233 | 237 | 238 |
| 25 | 217 | 220 | 222 | 233 | 244 | 249 | 250 |
| 26 | 227 | 230 | 232 | 243 | 256 | 260 | 261 |
| 27 | 237 | 240 | 242 | 254 | 266 | 271 | 272 |
| 28 | 246 | 249 | 251 | 264 | 277 | 282 | 282 |
| 29 | 255 | 258 | 260 | 273 | 287 | 291 | 292 |
| 30 | 263 | 266 | 269 | 282 | 296 | 300 | 301 |
| 31 | 271 | 274 | 276 | 290 | 304 | 309 | 310 |
| 32 | 277 | 281 | 284 | 297 | 312 | 316 | 317 |
| 33 | 284 | 287 | 290 | 303 | 319 | 323 | 324 |
| 34 | 289 | 293 | 296 | 309 | 325 | 328 | 330 |
| 35 | 294 | 298 | 301 | 313 | 330 | 332 | 335 |
| 36 | 297 | 302 | 306 | 316 | 334 | 335 | 339 |
| 37 | 300 | 306 | 310 | 319 | 337 | 336 | 342 |
| 38 | 302 | 308 | 313 | 320 | 338 | 337 | 343 |

**Supplementary Table S4:** Centiles of estimated fetal weight using the Hadlock formula (N=241). RECIPAL cohort, Southern Benin, 2014-2017.

| Week of gestation | 3th | 5th | 10th | 50th | 90th | 95th | 97th |
| --- | --- | --- | --- | --- | --- | --- | --- |
| 15 | 99 | 103 | 107 | 132 | 158 | 156 | 152 |
| 16 | 121 | 124 | 129 | 151 | 163 | 165 | 165 |
| 17 | 149 | 151 | 158 | 180 | 180 | 187 | 192 |
| 18 | 183 | 186 | 195 | 219 | 211 | 221 | 231 |
| 19 | 225 | 229 | 240 | 269 | 255 | 269 | 283 |
| 20 | 273 | 278 | 293 | 330 | 311 | 329 | 348 |
| 21 | 329 | 336 | 355 | 401 | 381 | 403 | 426 |
| 22 | 392 | 402 | 424 | 483 | 463 | 490 | 517 |
| 23 | 463 | 476 | 503 | 575 | 558 | 589 | 621 |
| 24 | 542 | 559 | 590 | 678 | 666 | 701 | 738 |
| 25 | 630 | 650 | 685 | 791 | 786 | 827 | 867 |
| 26 | 727 | 750 | 790 | 915 | 919 | 965 | 1008 |
| 27 | 832 | 860 | 904 | 1049 | 1064 | 1116 | 1163 |
| 28 | 946 | 979 | 1027 | 1194 | 1222 | 1280 | 1330 |
| 29 | 1071 | 1108 | 1160 | 1350 | 1393 | 1456 | 1509 |
| 30 | 1204 | 1246 | 1302 | 1516 | 1575 | 1646 | 1700 |
| 31 | 1348 | 1395 | 1454 | 1692 | 1770 | 1848 | 1905 |
| 32 | 1502 | 1554 | 1616 | 1879 | 1977 | 2063 | 2121 |
| 33 | 1667 | 1724 | 1788 | 2077 | 2196 | 2291 | 2349 |
| 34 | 1843 | 1905 | 1970 | 2285 | 2427 | 2532 | 2590 |
| 35 | 2029 | 2096 | 2162 | 2504 | 2671 | 2785 | 2843 |
| 36 | 2227 | 2299 | 2365 | 2733 | 2926 | 3051 | 3108 |
| 37 | 2437 | 2514 | 2578 | 2973 | 3193 | 3330 | 3385 |
| 38 | 2658 | 2740 | 2802 | 3223 | 3471 | 3622 | 3674 |

**Supplementary Table S5:** Centiles of estimated fetal weight using the INTERGROWTH-21st formula starting from 22 weeks of gestation (N=241). RECIPAL cohort, Southern Benin, 2014-2017.

| Week of gestation | 3th | 5th | 10th | 50th | 90th | 95th | 97th |
| --- | --- | --- | --- | --- | --- | --- | --- |
| 22 | 464 | 474 | 489 | 537 | 610 | 628 | 644 |
| 23 | 517 | 529 | 548 | 610 | 698 | 723 | 740 |
| 24 | 576 | 592 | 615 | 693 | 799 | 831 | 850 |
| 25 | 644 | 663 | 691 | 787 | 912 | 952 | 974 |
| 26 | 719 | 743 | 776 | 891 | 1038 | 1087 | 1113 |
| 27 | 803 | 832 | 869 | 1007 | 1178 | 1235 | 1268 |
| 28 | 897 | 932 | 973 | 1134 | 1331 | 1398 | 1439 |
| 29 | 1000 | 1042 | 1086 | 1273 | 1499 | 1575 | 1627 |
| 30 | 1114 | 1163 | 1210 | 1424 | 1681 | 1767 | 1833 |
| 31 | 1239 | 1297 | 1345 | 1588 | 1878 | 1974 | 2056 |
| 32 | 1375 | 1443 | 1491 | 1766 | 2091 | 2196 | 2298 |
| 33 | 1523 | 1602 | 1649 | 1956 | 2320 | 2433 | 2560 |
| 34 | 1683 | 1775 | 1819 | 2161 | 2565 | 2686 | 2841 |
| 35 | 1857 | 1963 | 2002 | 2379 | 2827 | 2955 | 3143 |
| 36 | 2044 | 2166 | 2198 | 2613 | 3106 | 3240 | 3465 |
| 37 | 2246 | 2384 | 2407 | 2861 | 3402 | 3541 | 3810 |
| 38 | 2462 | 2619 | 2631 | 3125 | 3716 | 3859 | 4176 |

**Supplementary Table S6:** Centiles of femur length (N=241). RECIPAL cohort, Southern Benin, 2014-2017.

| Week of gestation | 3th | 5th | 10th | 50th | 90th | 95th | 97th |
| --- | --- | --- | --- | --- | --- | --- | --- |
| 15 | 15 | 15 | 15 | 18 | 20 | 21 | 21 |
| 16 | 18 | 18 | 18 | 21 | 23 | 24 | 25 |
| 17 | 20 | 21 | 22 | 24 | 26 | 27 | 28 |
| 18 | 23 | 24 | 25 | 27 | 29 | 30 | 31 |
| 19 | 26 | 27 | 28 | 30 | 32 | 33 | 34 |
| 20 | 29 | 30 | 30 | 33 | 35 | 36 | 37 |
| 21 | 32 | 33 | 33 | 35 | 38 | 39 | 40 |
| 22 | 35 | 35 | 36 | 38 | 41 | 42 | 43 |
| 23 | 37 | 38 | 38 | 41 | 44 | 44 | 45 |
| 24 | 40 | 40 | 41 | 43 | 46 | 47 | 48 |
| 25 | 42 | 43 | 43 | 46 | 49 | 50 | 51 |
| 26 | 45 | 45 | 46 | 48 | 52 | 52 | 53 |
| 27 | 47 | 47 | 48 | 51 | 54 | 55 | 56 |
| 28 | 49 | 49 | 50 | 53 | 57 | 57 | 58 |
| 29 | 51 | 52 | 52 | 55 | 59 | 60 | 61 |
| 30 | 53 | 54 | 54 | 58 | 61 | 62 | 63 |
| 31 | 55 | 56 | 56 | 60 | 64 | 64 | 65 |
| 32 | 57 | 58 | 58 | 62 | 66 | 66 | 67 |
| 33 | 58 | 59 | 60 | 64 | 67 | 68 | 69 |
| 34 | 60 | 61 | 62 | 66 | 69 | 70 | 71 |
| 35 | 62 | 63 | 63 | 67 | 71 | 72 | 73 |
| 36 | 63 | 64 | 65 | 69 | 72 | 73 | 74 |
| 37 | 65 | 65 | 66 | 71 | 73 | 74 | 75 |
| 38 | 67 | 67 | 67 | 72 | 75 | 75 | 76 |

**Supplementary Table S7:** The proportion of fetuses with observed values below the threshold of each percentile (Q) using quantile regression to model RECIPAL data, RECIPAL cohort, Southern Benin, 2014-2017.

| **Abdominal Circumference** | | |
| --- | --- | --- |
| Q3 and Q97 | Proportion < 3% n / N (%) | 26/964 (2.7%) |
|  | Proportion < 97% n / N (%) | 934/964 (96.9%) |
| Q5 and Q95 | Proportion < 5% n / N (%) | 47/964 (4.9%) |
|  | Proportion < 95% n / N (%) | 914/964 (9.5%) |
| Q10 and Q90 | Proportion < 10% n / N (%) | 94/964 (9.8%) |
|  | Proportion < 90% n / N (%) | 865/964 (89.7%) |
| Q25 and Q75 | Proportion < 25% n / N (%) | 236/964 (24.5%) |
|  | Proportion < 75% n / N (%) | 721/964 (74.8%) |
| Q50 | Proportion < 50% n / N (%) | 475/964 (49.3%) |

| **Head Circumference** | | |
| --- | --- | --- |
| Q3 and Q97 | Proportion < 3% n / N (%) | 26/964 (2.7%) |
|  | Proportion < 97% n / N (%) | 933/964 (96.8) |
| Q5 and Q95 | Proportion < 5% n / N (%) | 47/964 (4.9%) |
|  | Proportion < 95% n / N (%) | 912/964 (94.6%) |
| Q10 and Q90 | Proportion < 10% n / N (%) | 94/964 (9.8%) |
|  | Proportion < 90% n / N (%) | 865/964 (89.7%) |
| Q25 and Q75 | Proportion < 25% n / N (%) | 235/964 (24.4%) |
|  | Proportion < 75% n / N (%) | 721/964 (74.8%) |
| Q50 | Proportion < 50% n / N (%) | 477/964 (49.5%) |

| **Femur Length** | | |
| --- | --- | --- |
| Q3 and Q97 | Proportion < 3% n / N (%) | 27/964 (2.8%) |
|  | Proportion < 97% n / N (%) | 933/964 (96.8) |
| Q5 and Q95 | Proportion < 5% n / N (%) | 46/964 (4.8%) |
|  | Proportion < 95% n / N (%) | 912/964 (94.6) |
| Q10 and Q90 | Proportion < 10% n / N (%) | 93/964 (9.6%) |
|  | Proportion < 90% n / N (%) | 866/964 (89.8%) |
| Q25 and Q75 | Proportion < 25% n / N (%) | 238/964 (24.7) |
|  | Proportion < 75% n / N (%) | 721/964 (74.8%) |
| Q50 | Proportion < 50% n / N (%) | 479/964 (49.7%) |

| **Estimated fetal weight (using Hadlock formula)** | | |
| --- | --- | --- |
| Q3 and Q97 | Proportion < 3% n / N (%) | 27/964 (2.8%) |
|  | Proportion < 97% n / N (%) | 933/964 (96.8) |
| Q5 and Q95 | Proportion < 5% n / N (%) | 46/964 (4.8%) |
|  | Proportion < 95% n / N (%) | 914/964 (94.8) |
| Q10 and Q90 | Proportion < 10% n / N (%) | 95/964 (9.9%) |
|  | Proportion < 90% n / N (%) | 865/964 (89.7%) |
| Q25 and Q75 | Proportion < 25% n / N (%) | 240/964 (24.9%) |
|  | Proportion < 75% n / N (%) | 721/964 (74.8%) |
| Q50 | Proportion < 50% n / N (%) | 480/964 (49.8%) |

| **Estimated fetal weight (using INTERGROWTH-21st formula)** | | |
| --- | --- | --- |
| Q3 and Q97 | Proportion < 3% n / N (%) | 27/964 (2.8%) |
|  | Proportion > 97% n / N (%) | 933/964 (96.8) |
| Q5 and Q95 | Proportion < 5% n / N (%) | 47/964 (4.9%) |
|  | Proportion > 95% n / N (%) | 914/964 (94.8%) |
| Q10 and Q90 | Proportion < 10% n / N (%) | 93/964 (9.6%) |
|  | Proportion > 90% n / N (%) | 866/964 (89.8%) |
| Q25 and Q75 | Proportion < 25% n / N (%) | 240/964 (24.9%) |
|  | Proportion > 75% n / N (%) | 725/964 (75.2%) |
| Q50 | Proportion < 50% n / N (%) | 480/964 (49.8%) |
