# Supplementary material for: Assessing fetal growth in Africa: Application of the international WHO and INTERGROWTH-21st standards in a Beninese pregnancy cohort
Source: PLoS One. 2022 Jan 21;17(1):e0262760. doi: 10.1371/journal.pone.0262760 (PMC8782373; doi:10.1371/journal.pone.0262760)
Supplement: Attachment — Submitted filename: Response to Reviewers.docx [file pone.0262760.s008.docx]

**Dear editor,**

Thank you for considering our manuscript PONE-D-21-11431R1: Assessing fetal growth in Africa: application of the international WHO and INTERGROWTH-21st standards in a Beninese pregnancy cohort. We have revised the issues brought up by the reviewers and a point-by-point response can be found below.

As requested by the reviewers, we have made the minor revisions requested: The proposed rewordings have been taken into account and reference 29 has been quoted in the right place.

With kind regards,

Dr Emmanuel Koffi YOVO

Clinical Research Institute of Benin (IRCB)

04 BP 1114, Abomey-Calavi

Email: emkoffiyovo@gmail.com

Tel: +229 97 42 08 01

| **Response to reviewers** | |
| --- | --- |
| **Comments** | **Responses** |
| **Response to Reviewer # 2** | |
| The comments were addressed but Table 2 needs to be modified. Confusing. it is written male, The text is unclear birth weight = should be newborn weight .... 2500gm ? | We thank the reviewer for the comment regarding the table 2. We have reorganised the table 2 with more details that make it easier to understand  We have referred to birth weight as the weight measured within minutes of the birth of each child. |
| **Response to Reviewer # 3** | |
| 1. Authors' revision has addressed main point | We thank the reviewer for the positive comments about the revised version of the paper. |
| 2. Coefficient for Altman Bland plot ought to be added. | Coefficients are shown in Figures 7 and 8, and Supplementary Figures 4 and 5 |
| 3. Inclusion of new version of Intergrowth formula (using Hadlock for EFW) is a good step as it makes results 'current' with formula changes. However, note that relevant reference for this correction by IG21 is 29, not 28. | Done,  Thank you very much for your attention which made it possible to correct the numbering error |
| 4. Lines 256-7: IG21hl 'did not do better' is wrong term as there are no outcomes to use as a performance standard. Perhaps 'performed similarly' or 'had similar discrepancy to WHO' (as IG21) | Done,  Thank you for the rewording suggestions which are taken into account |
| 5. Lines 331-2 As in 4, as no standard used/available, 'better match' needs to be reworded; perhaps 'seemed to better reflect...' | Done,  Thank you for the rewording suggestions which are taken into account |
